# The Effect of Pre-retirement Preparation on Post-retirement Well-being: The Mediating Role of Sense of Control

**DOI:** 10.1093/geroni/igaf122.3857

**Published:** 2025-12-31

**Authors:** Gloria Lin, Dannii Yeung, Edwin Ka, Hung Chung

**Affiliations:** City University of Hong Kong, Hong Kong, China (People’s Republic); City University of Hong Kong, Hong Kong, China (People’s Republic); City University of Hong Kong, Hong Kong, China (People’s Republic); City University of Hong Kong, Hong Kong, China (People’s Republic)

## Abstract

Previous research has established the linkage between pre-retirement preparation and post-retirement well-being (Yeung, 2018), but the underlying mechanisms have not been fully explored. Drawing on Social Cognitive Theory (Bandura, 1978), this study examined the mediating effect of sense of control on this relationship in 943 Hong Kong Chinese retirees (Mage = 71.76 years, SD = 5.28, range = 65 – 95). Using a cross-sectional design, retirees’ retirement preparation, sense of control (i.e., perceived constraints [PC] and personal mastery [PM]), life satisfaction, positive and negative affect, and depressive symptoms were assessed. Results demonstrated a significant main effect of retirement preparation on all well-being measures. Both PC and PM mediated the effect of retirement preparation on life satisfaction, negative affect, and depressive symptoms, while only PM mediated the effect on positive affect. Specifically, retirees with more retirement preparation exhibited greater PM and fewer PC, resulting in higher life satisfaction (β = .02, 95%CI = .00, .03 for PC; β = .03, 95%CI = .01, .06 for PM), more positive emotions (β = .02, 95%CI = .01, .04 for PM), and fewer negative emotions (β = –.03, 95%CI = –.06, –.00 for PC; β = –.01, 95%CI = –.02, –.00 for PM) and depressive symptoms (β = –.03, 95%CI = –.05, –.01 for PC; β = –.02, 95%CI = –.03, –.01 for PM). The results remained significant even after controlling age, gender, and socioeconomic status. Our findings underscored the protective role of retirement preparation in sustaining sense of control and post-retirement psychological well-being.

